# Supplementary material for: The balance between B55α and Greatwall expression levels predicts sensitivity to Greatwall inhibition in cancer cells
Source: Nat Commun. 2025 Aug 27;16:8016. doi: 10.1038/s41467-025-62943-z (PMC12391391; doi:10.1038/s41467-025-62943-z)
Supplement: Supplementary file 1 — Supplementary Information [file 41467_2025_62943_MOESM1_ESM.pdf]

Supplementary Figure 1

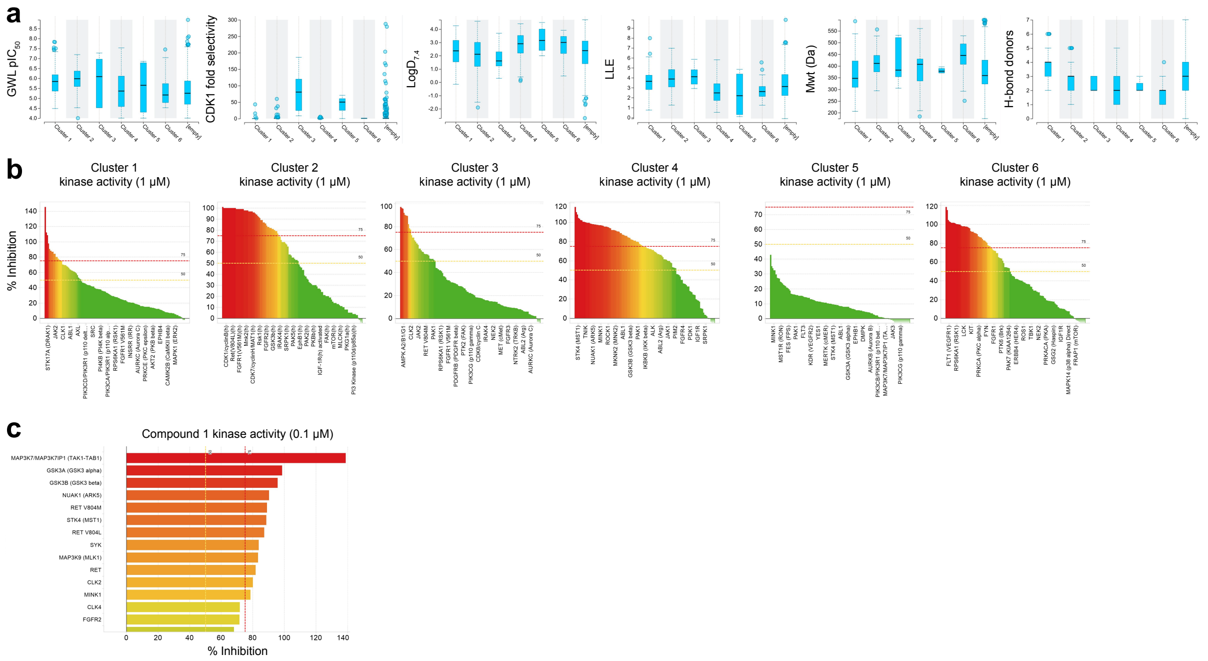

Supplementary Fig 1. | Summary of screening output and the 6 priority compound clusters. a Analysis of the 6 priority clusters against key triage properties including GWL pIC_{50}, fold selectivity vs Cdk1, lipophilicity ($logD_{7.4}$), ligand lipophilicity efficiency (LLE; GWL pIC_{50} - $LogD_{7.4}$), molecular weight (Mwt) and number of hydrogen bond (H-bond) donors. [Empty] series refers to properties of the non-prioritised hits. **b** Kinome per cent inhibition (% inhibition) selectivity data for a single exemplar from each of the 6 priority clusters. **c** Identity of kinases with >75% inhibition (0.1 µM) for Compound **1** from Cluster 3.

Supplementary Figure 2

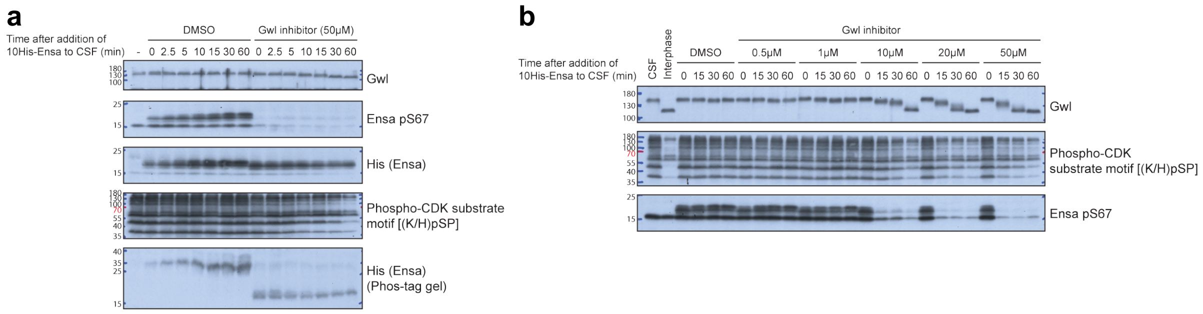

Supplementary Fig 2. | Characterisation of C-604 efficiency in *Xenopus laevis egg* extracts. a, b Western blots (n = 1) demonstrating the impact of C-604 on ENSA(S67) and GWL phosphorylation levels in *X. laevis* egg extracts.

Supplementary Figure 3

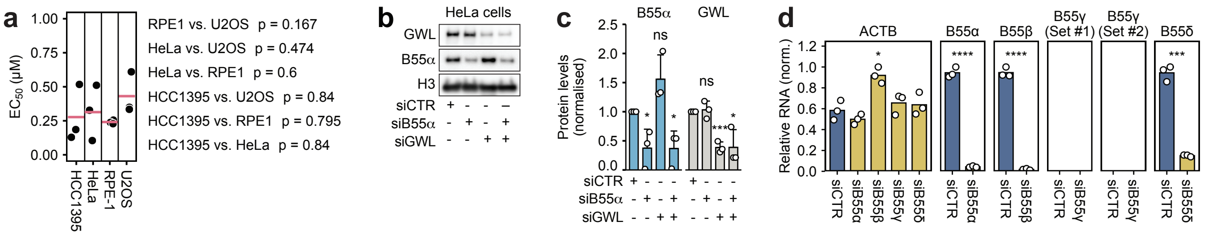

Supplementary Fig 3. | Additional characterisation of C-604 efficiency in human cells. a Comparison of cellular C-604 EC_{50} values determined in HCC1395, HeLa, RPE-1 and U2OS cells. Individual points represent outcomes of n = 3 independent experiments. Horizontal red lines represent means. Statistical significance was determined by an unpaired two-tailed t-test. Parametric statistical testing was justified by the Shapiro-Wilk test of normality ($p \geq 0.11$). Determined p-values are indicated. **b** Representative western blot showing GWL and B55α expression levels in HeLa cells 24 hours post-transfection with siRNAs targeting GWL (siGWL) or B55α (siB55α). Histone H3 was used as a loading control. **c** GWL and B55α protein levels relative to the loading control following the transfection with siRNAs targeting GWL or B55α. Relative GWL or B55α protein levels are normalised to the respective expression in cells transfected with control siRNA (siCTR). Bars and error bars indicate the means and standard deviations of n = 3 independent measurements. Statistical significance was determined using an unpaired two-tailed t-test. **d** Relative RNA expression levels of B55 isoforms in RPE-1 cells 24 hours post-transfection with siRNAs specific to B55α, B55β, B55γ and B55δ. RNA expression levels relative to GAPDH were normalised to the maximum value. RNA expression of ACTB was used as a control. Bars represent means of n = 3 independent measurements (individual points). Statistical significance was determined using an unpaired two-tailed t-test. Parametric statistical testing was justified by Shapiro-Wilk test of normality ($p \geq 0.36$). Determined p-values were corrected for multiple testing using the Benjamini-Hochberg procedure. ns – not significant, * $p < 0.05$, ** $p < 0.01$, *** $p < 0.001$, **** $p < 0.0001$.

Supplementary Figure 4

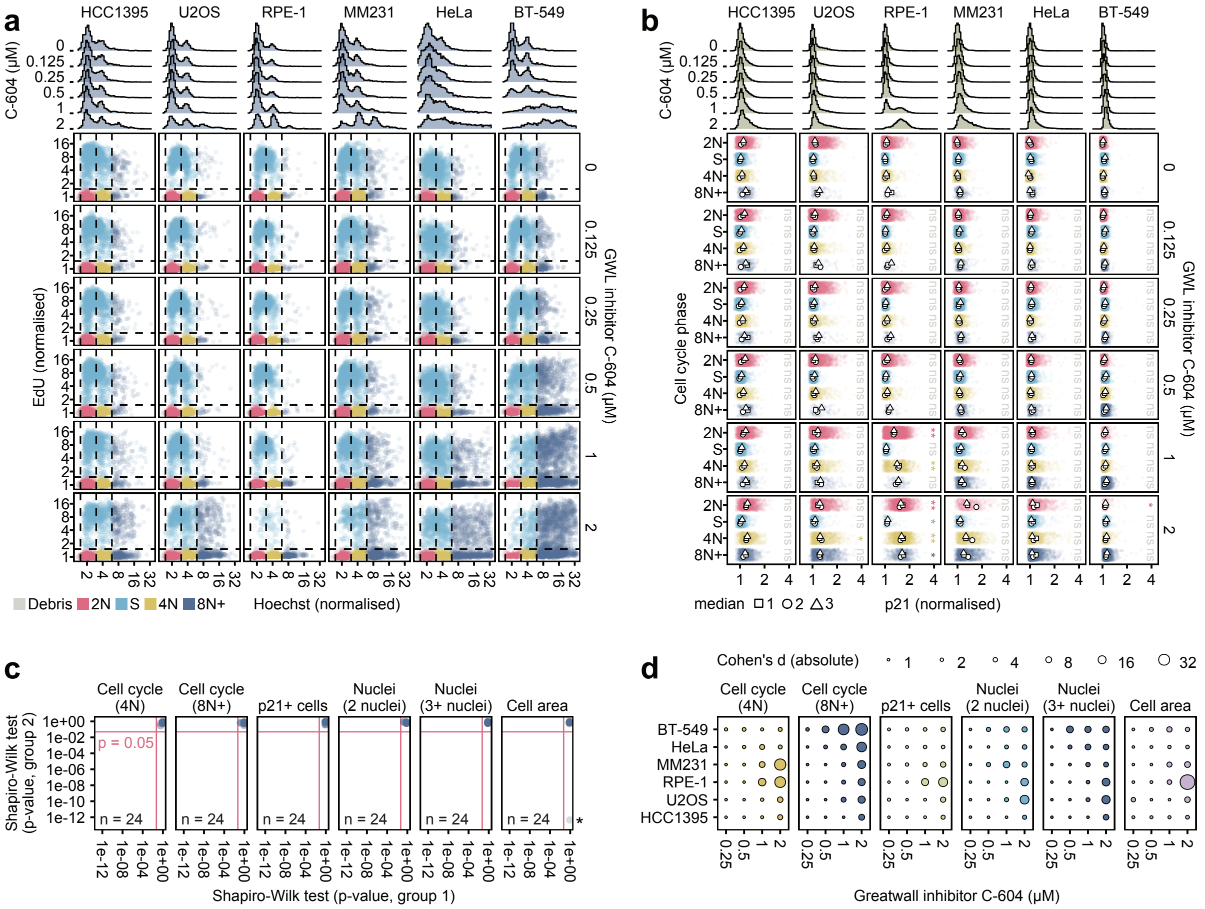

Supplementary Fig. 4 | Single-cell analysis of cellular responses to GWL inhibition by C-604. a Scatter plots of single-cell Hoechst and EdU intensities in indicated cell lines treated with C-604 for 72 h. Dashed lines represent boundaries between distinct, colour-coded cell cycle groups. Histograms represent distributions of Hoechst intensities. Pooled data from three independent repeats are shown. The Maximum number of displayed events per dataset is 2,000. **b** Strip plots of single-cell p21 intensities in different cell cycle groups of C-604-treated cells. Dots, squares and triangles represent medians of n = 3 independent repeats. Histograms represent distributions of p21 intensities. Scatterplots and strip plots display the pooled results from three independent experiments with up to 2,000 randomly selected cells per condition for each experiment. Statistical significance was determined from median values using an unpaired two-tailed t-test, ns – not significant, * $p < 0.05$, ** $p < 0.01$, *** $p < 0.001$. Determined p-values were adjusted for multiple testing using the Benjamini-Hochberg procedure. Parametric statistical testing was justified by the Shapiro-Wilk test of normality ($p > 0.07$). **c** Results of Shapiro-Wilk test relevant to the analysis presented in (Fig. 2d). Out of 24 sample comparisons in 6 distinct datasets, one (*) did not satisfy the normal distribution (MM231, cell area, C-604 = 0.5 μM). **d** Size effect analysis of data presented in (Fig. 2d). Absolute Cohen's d values are shown.

Supplementary Figure 5

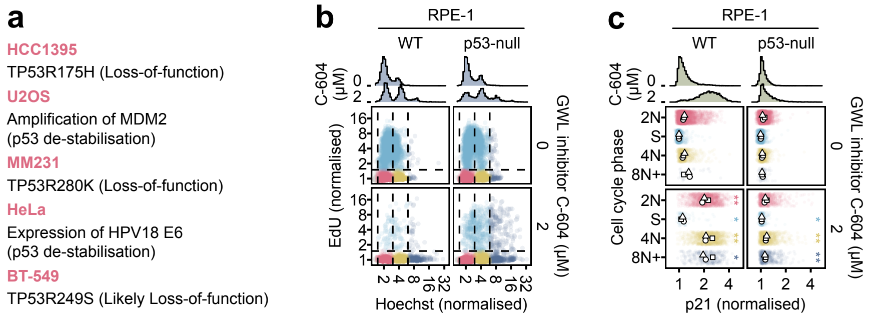

Supplementary Fig. 5 | Extended analysis of C-604-induced pathologies in WT and p53-null RPE-1 cells. a Genetic characteristics negatively affecting p53 function in the indicated cancer cell line models. **b** Scatter plots of single-cell Hoechst and EdU intensities in WT and p53-null RPE-1 cells treated with C-604 for 72 h. Histograms represent distributions of Hoechst intensities. **c** Strip plots of single-cell p21 intensities in distinct cell cycle groups of C-604 treated WT and p53-null cells. Dots, squares and triangles represent medians of n = 3 independent repeats. Histograms represent distributions of p21 intensities. Scatterplots and strip plots display the pooled results from three independent experiments with up to 2000 randomly selected cells per condition for each experiment. Statistical significance of differences between median p21 intensities of untreated (0 μM C-604) and treated (2 μM C-604) cells was determined using an unpaired two-tailed t-test, ns – not significant, * $p < 0.05$, ** $p < 0.01$, *** $p < 0.001$. Determined p-values were adjusted for multiple testing using the Benjamini-Hochberg procedure. Parametric statistical testing was justified by the Shapiro-Wilk test of normality ($p > 0.34$).

Supplementary Figure 6

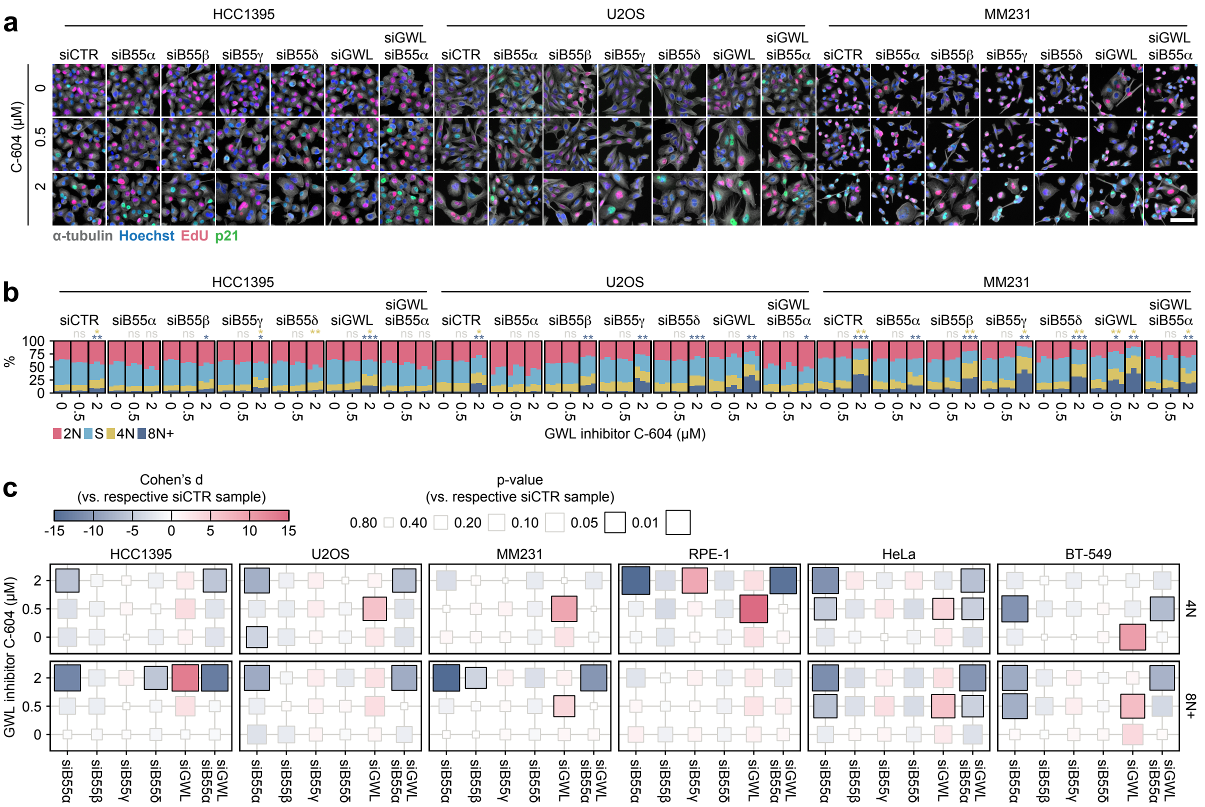

Supplementary Fig. 6 | The impact of siRNA-mediated depletion of GWL and B55α-δ on cellular responses to GWL inhibition by C-604 in additional cell lines. a Representative images HCC1395, U2OS and MM231 cells treated with siRNAs targeting B55α, B55β, B55γ, B55δ or GWL exposed to C-604 for 48 h. Images from one of n = 3 independent experiments are shown. The scale bar represents 50 µm. **b** Distributions of cell cycle groups in control and C-604-treated populations depleted of B55α-δ or GWL. The results of n = 3 independent experiments are shown for each condition as separate columns. The cell cycle state in the stacked bar plot and indication of statistical significance are colour-coded as specified by the colour legend below the plots. Between 885 (Experiment 1, MM231, siGWL, 2 µM C-604) and 15,112 (Experiment 1, HCC1395, siCTR, 0 µM C-604) cells were analysed. Statistical significance of differences between proportions of 4N and 8N+ cells was determined by an unpaired two-tailed t-test. Parametric statistical testing was justified by the Shapiro-Wilk test of normality ($p \geq 0.74$). Determined p-values were adjusted for multiple testing using the Benjamini-Hochberg procedure. ns – not significant, * $p < 0.05$, ** $p < 0.01$, *** $p < 0.001$. **c** Comparison of the effects of the specified siRNAs on the proportions of 4N and 8N+ cells, relative to their respective siCTR controls. Effect sizes were quantified using Cohen's d. Statistical significance was assessed using an unpaired two-tailed t-test, with p-values adjusted for multiple comparisons via the Benjamini-Hochberg procedure.

Supplementary Figure 7

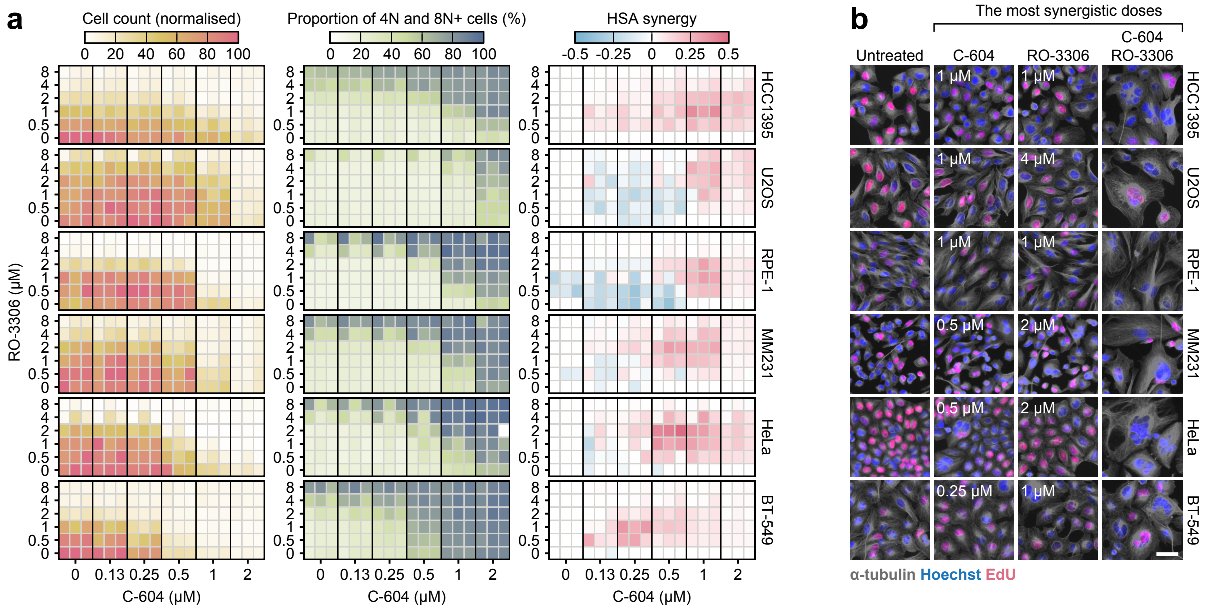

Supplementary Fig. 7. | Characterisation of the functional synergy between C-604 and RO-3306 treatments. a Cell counts, combined proportions of 4N and 8N+ cells and highest single agent (HSA) synergy values in populations of indicated cell lines treated with increasing concentrations of C-604 and RO-3306 for 72 h. For each experiment and cell line, cell counts were normalised to the maximum recorded value. The HSA values were derived from normalised cell counts using the formula: HSA = $E_{CR} - \max(E_C, E_R)$, where E_{CR} denotes the combined effect of C-604 and RO-3306, and E_C and E_R represent the individual effects of C-604 and RO-3306, respectively. Results of three independent experiments are shown as three separate columns per condition. Between 6 (Experiment 3, HeLa, 2 µM C-604, 1 µM RO-3306) and 43,987 (Experiment 3, HeLa, 0.125 µM C-604, 0.5 µM RO-3306) cells were analysed. **b** Representative images of cells treated with the most synergising doses of C-604 and RO-3306. The respective doses of C-604 and RO-3306 are indicated. The scale bar represents 50 µm.

Supplementary Figure 8

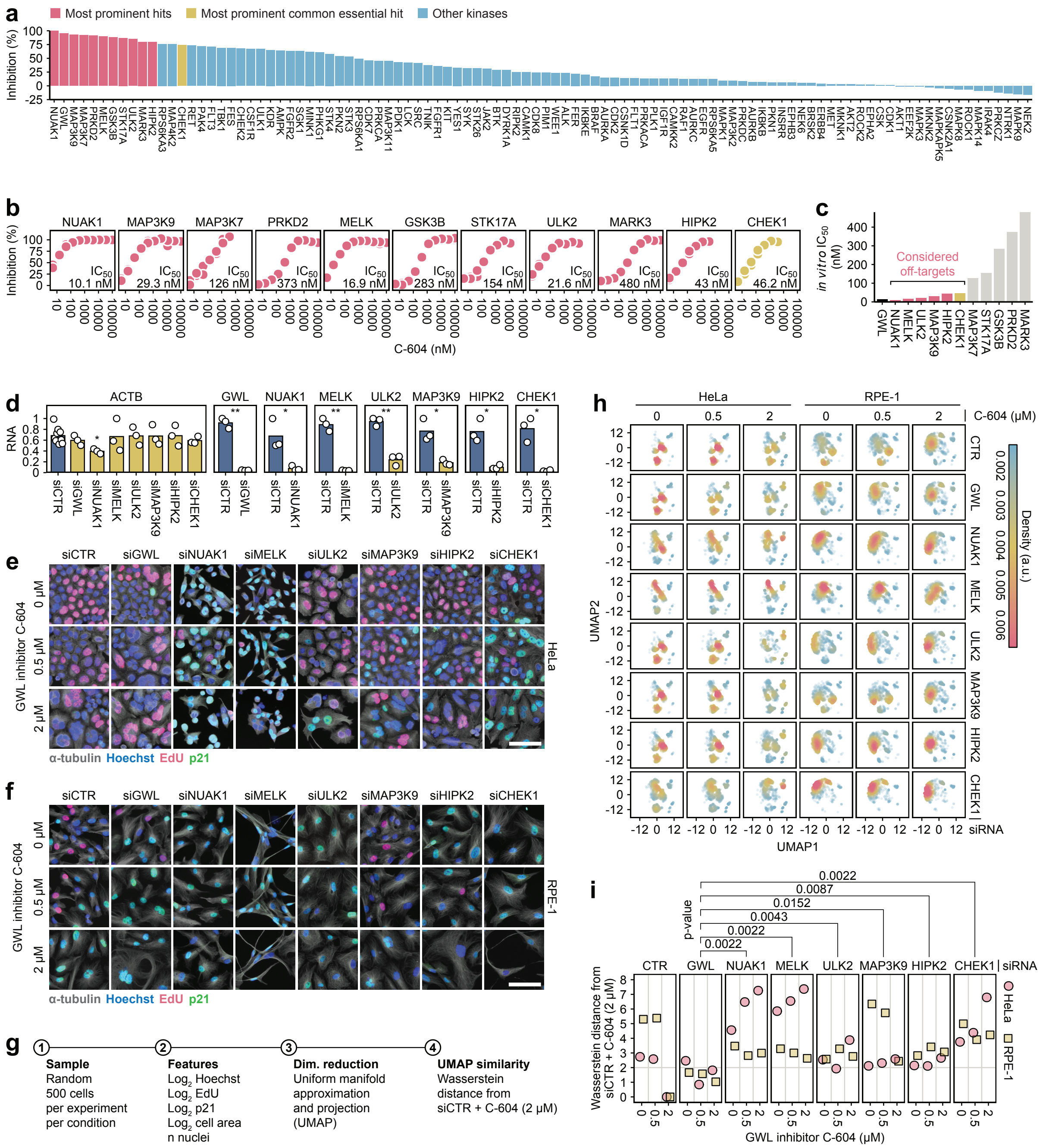

Supplementary Fig. 8 | Experimental evaluation of the potential off-target activity of C-604. a The impact of C-604 (1 µM) on the *in vitro* activity of 100 kinases. Targets inhibited by at least 80% are labelled red. The most prominent common essential hit is labelled yellow. Other kinases are labelled blue. **b** *In vitro* dose-dependent C-604-mediated inhibition of suspected off-target kinases. Established *in vitro* IC_{50} values are indicated. **c** Ordered *in vitro* IC_{50} values of suspected C-604 off-targets alongside HTRF-derived IC_{50} of GWL. Lead off-target candidates (IC_{50} ≤ 50 nM) are indicated. **d** RT-qPCR analysis of relative RNA levels of GWL and suspected C-604 off-target kinases. Points represent results of n = 3 independent experiments. Bars represent means. Each gene-specific RNA expression was normalised to GAPDH. Expression of ACTB was used as a negative control. Statistical significance was determined using an unpaired two-tailed t-test, ns – not significant, * $p < 0.05$, ** $p < 0.01$. Parametric statistical testing was justified by the Shapiro-Wilk test of normality ($p > 0.27$). All reported p-values were adjusted for multiple testing using the Benjamini-Hochberg procedure. **e, f** Representative images of C-604-treated HeLa (e) and RPE-1 (f) cells transfected with siRNAs targeting GWL or suspected off-

target kinases. The scale bar represents 50 µm. **g** Step-by-step illustration of the phenotypic trait comparison analysis involving data sampling, feature extraction, dimensionality reduction by uniform manifold approximation and projection (UMAP) and UMAP similarity assessment based on Wasserstein distances. **h** UMAP profiles of C-604-treated HeLa and RPE-1 cells transfected with siRNAs specific to suspected off-target kinases. Pooled data from n = 3 independent experiments are shown (3 × 500 cells per condition). **i** Wasserstein distances of distinct UMAP profiles from the UMAP representation of HeLa or RPE-1 populations transfected with the control siRNA (siCTR) and treated with 2 µM C-604. Low Wasserstein distances indicate similarity. High Wasserstein distances indicate deviation from the phenotypic responses consequential to 2 µM C-604. Statistical significance was determined using the non-parametric unpaired Wilcoxon test. Determined p-values are shown.

Supplementary Figure 9

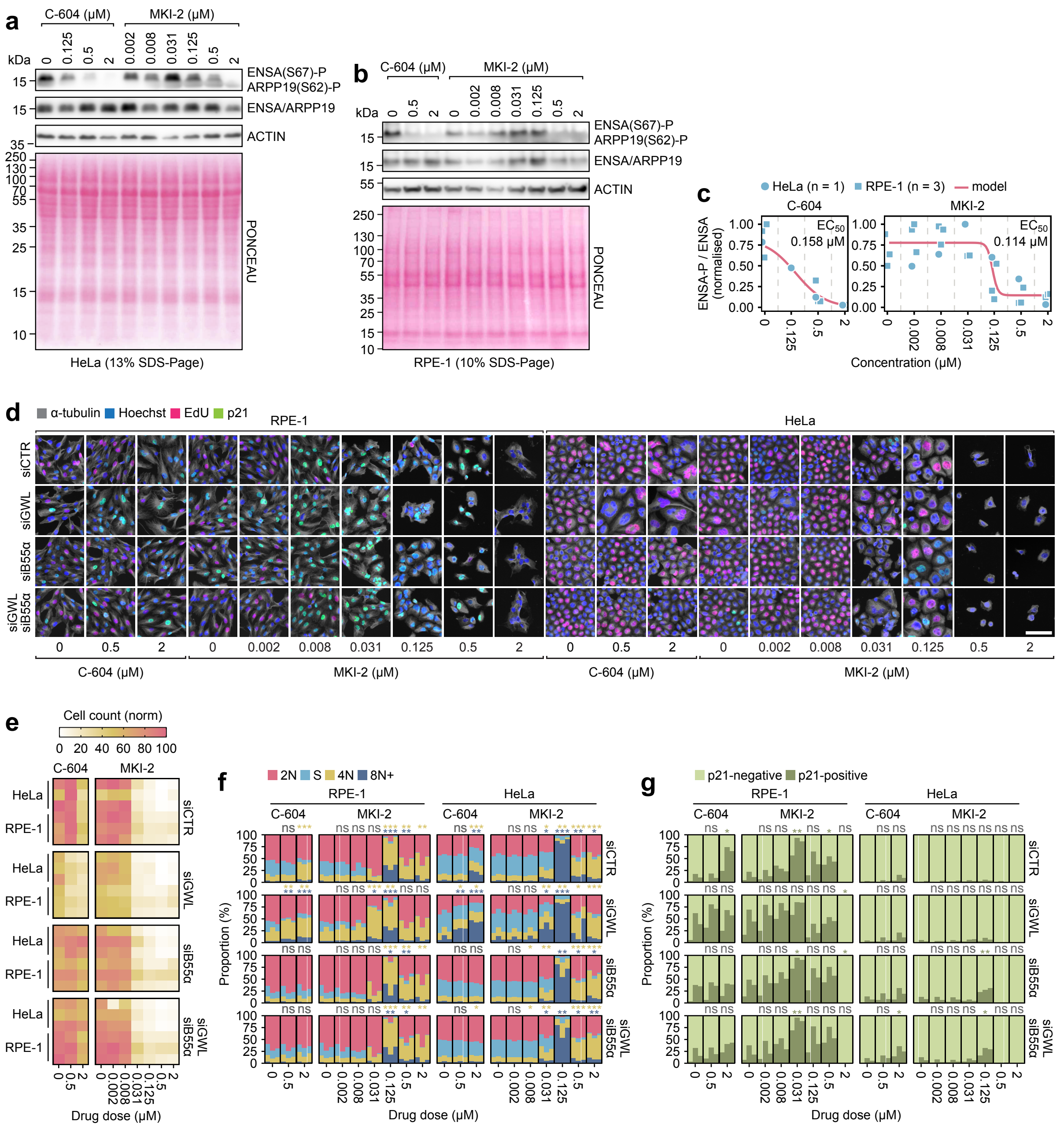

Supplementary Fig. 9. | Biological characterisation of the alternative GWL inhibitor MKI-2. a, b Representative western blot images showing changes in ENSA(S62)/ARPP19(S67) phosphorylation levels in prometaphase-arrested HeLa (a) and RPE-1 (b) cells exposed to increasing concentrations of C-604 or MKI-2 for 30 min. The experiment was performed n = 1 time for HeLa cells and n = 3 times for RPE-1 cells. Actin beta was used as a loading control. **c** Quantification of the impact of C-604 or MKI-2 on ENSA(S62)/ARPP19(S67) phosphorylation levels in prometaphase-arrested HeLa (n = 1) and RPE-1 (n = 3) cells. Circles and squares represent individual measurements. Solid red line represents a four-parameter sigmoidal model fitted using the least squares estimation method. Determined EC_{50} values are indicated. **d** Representative immunofluorescence images of RPE-1 and HeLa cells transfected with siRNA targeting GWL or B55α treated with C-604 or MKI-2 for 72 hours. Images from one of n = 3 independent experiments are shown. Scale bar represents 50 μm. **e** Normalised cell counts of RPE-1 and HeLa cells grown under the indicated conditions. The results of three independent experiments are shown as independent rows. In each experiment, cell counts were normalised to the maximum count recorded for each cell line. **f, g** Distributions of cell cycle groups (f) and proportions of quiescent/senescent p21+ cells (g) in cell populations cultured under indicated conditions. The results

of n = 3 independent experiments are shown for each condition as separate columns. Between 4 (experiment 3, HeLa, siGWL, 0.5 µM MKI-2) and 61,040 (experiment 3, HeLa, siCTR, 0.002 µM MKI-2) cells were analysed. Statistical significance was determined using an unpaired two-tailed t-test from the shown proportions (%). Parametric statistical testing was justified by the Shapiro-Wilk test of normality (cell cycle analysis: 127/128 tested samples $p > 0.05$; p21 expression analysis: 58/64 tested samples $p > 0.05$). ns – not significant, * $p < 0.05$, ** $p < 0.01$, *** $p < 0.001$.

Supplementary Figure 10

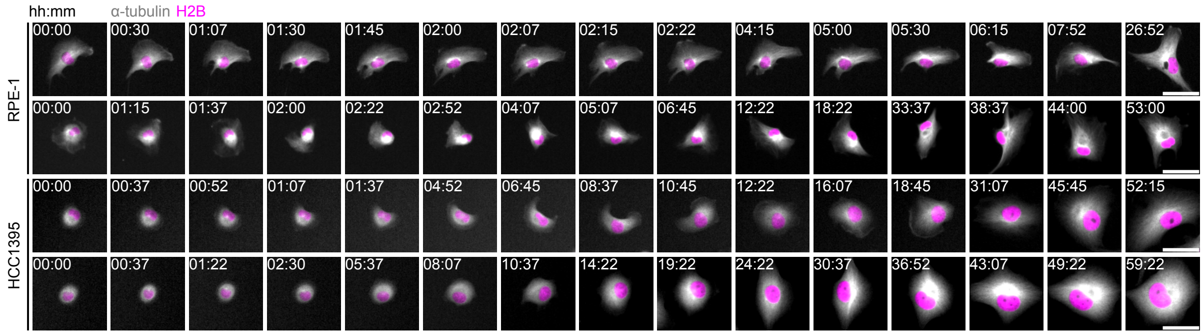

Supplementary Fig. 10 | Mitotic slippage events with subtle signs of G2/M transition. Representative image sequences of live RPE-1 and HCC1395 cells expressing α-tubulin-GFP and H2B-mCherry treated with 2 μM C-604. Indicated cells display minor signs of mitotic commitment, such as cell rounding, centrosome growth and centrosome separation, followed by a reversal to interphase. Displayed image sequences are from one of n = 3 independent experiments.

Supplementary Figure 11

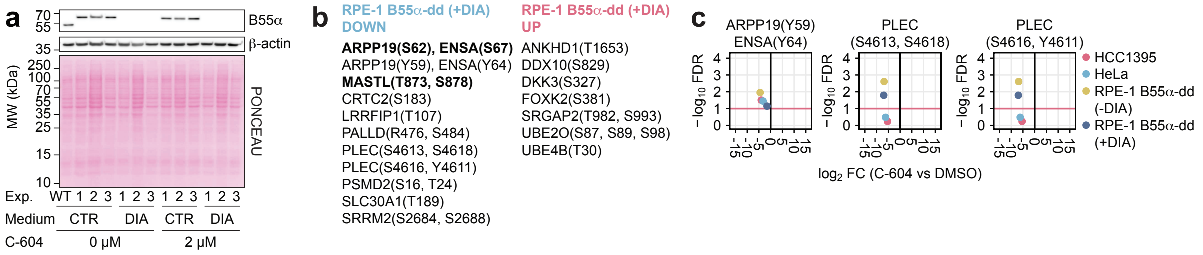

Supplementary Fig. 11 | A supplement to the phosphoproteomic analysis of cellular responses to C-604-induced GWL inhibition. a Western blot demonstrating DIA-induced degradation of B55α in RPE-1 prometaphase-arrested B55α-dd cells used for the mass-spectrometry analysis presented in (Fig. 5). B55α expression levels of three independently collected samples are shown. β-actin was used as a loading control. DIA – doxycycline, indole-3-acetic acid, Asunaprevir. **b** List of phosphosites deregulated (up or down) in RPE-1 B55α-dd (+DIA) cells. **c** Changes in phosphorylation levels of indicated phosphosites in C-604-treated prometaphase-arrested cells. The horizontal red line represents the FDR threshold (FDR > 0.1) of statistical significance.

Supplementary Figure 12

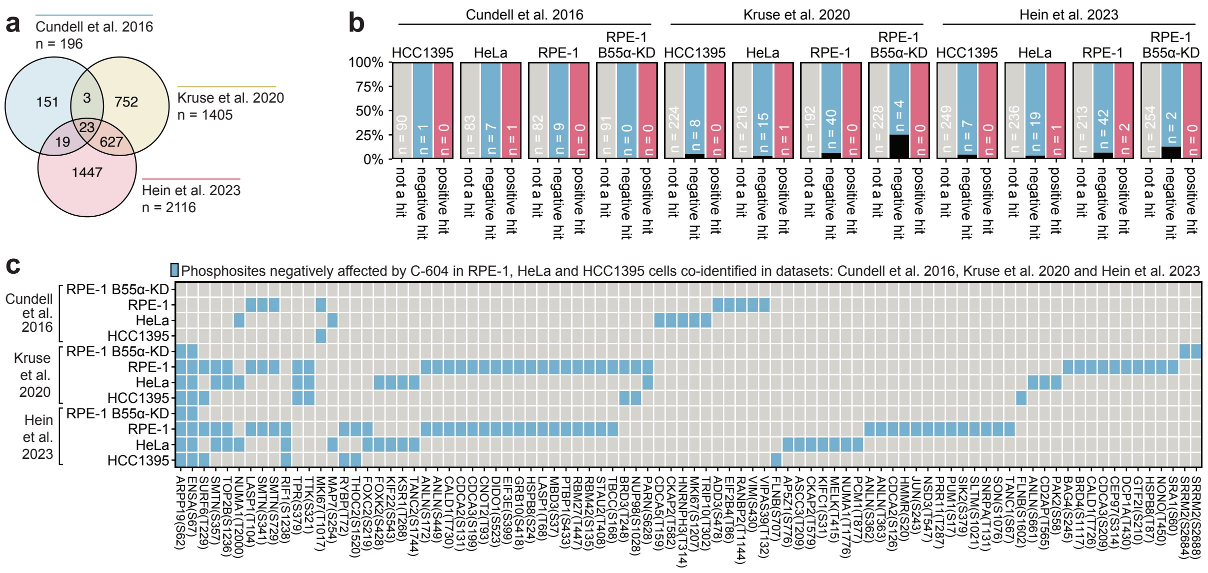

Supplementary Fig. 12 | Comparative intersection analysis of phosphosites impacted by C-604 and published PP2A-B55 substrates. a An intersection analysis of three publicly available datasets of PP2A-B55 substrates[1–3]. **b** Numbers of phosphosites impacted by C-604 co-identified in the indicated publicly available sets of PP2A-B55 substrates. Black bars represent the percentage of co-identified hits. **c** Representation of C-604-sensitive phosphosites co-identified in the indicated datasets.

Supplementary Table 1 | Compound **1** profiling

	Profiling study	Compound **1**
Potency	^{K72M}GWL HTRF pIC_{50}	7.0
Off-target	CDK1 pIC_{50}	5.5
	Syk pIC_{50}	7.6
	hERG %inhibition (pIC_{50})	32% (<4.5)
LLE	LLE	3.3
Phys-Chem Properties	Mwt	305
	LogD $pH_{7.4}$	3.7
	PSA (Å^2)	68
	HBD / HBA	2 / 6
	Solubility (µM)	0.2
In vitro DMPK	hMic Clint (µL/min/mg)	45
	rHep Clint (µL/min/mg)	261

Supplementary Table 2 | Inhibition of MASTL activity for pyrrolopyrazines **1**, **6** - **21**

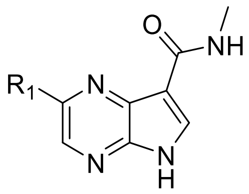

Compound	R_1	MASTL IC_{50} μM	cLogD**	Clint (Mouse) (μL/min/mg)	Solubility (μM)
**1**	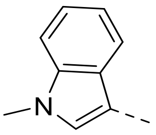	0.109*	-	-	-
**6**	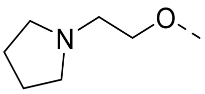	5.885#	-	-	-
**7**	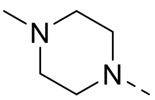	4.242#	-	-	-
**8**	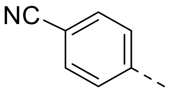	0.339#	-	-	-
**10**	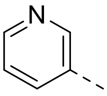	0.507#	-	-	-
**11**	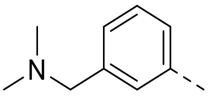	0.206#	-	-	-
**12**	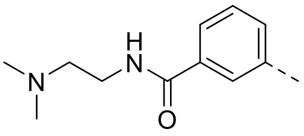	0.062#	-	-	-
**13**	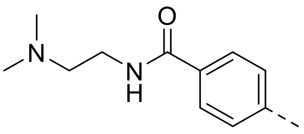	0.094#	-	-	-
**14**	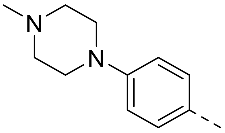	0.048	-	-	-
**15**	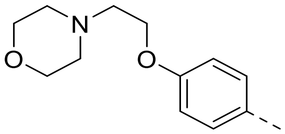	0.191#	1.08	11	-

**16**	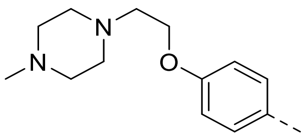	0.031#	0.64	18	-
**17**	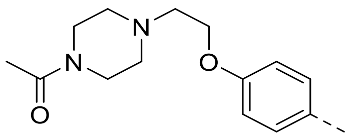	0.080#	0.35	7	-
**18**	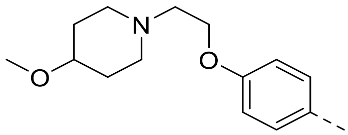	0.041	0.12	20 (Rat)	504
**19**	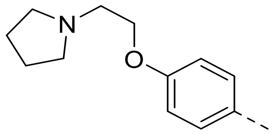	0.030	0.25	37	-
**20**	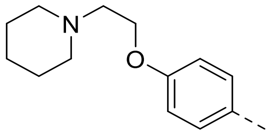	0.017	1.08	12	585
**21**	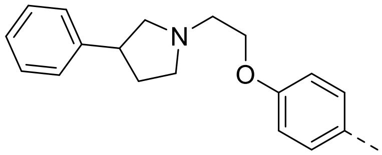	0.013	1.5	24	72

* Batch 1 WT MASTL
# K72M MASTL
** Chemaxon

Supplementary Table 3 | Profiling of selected compounds.

Profiling study	Compound **1**	Compound **14**	Compound **21**
IC_{50} MASTL (µM)	0.109	0.048	0.013
IC_{50} CDK1/cyclinB (µM)	1.477	>30	>30
Solubility pH 7.0 (µM)	15	260	72
Cl (mouse) (µL/min/mg)	196	83	24
Cl (human) (µL/min/mg)	147	9	13
Caco-2 AB/BA (10^{-6} cm/sec)	17/25	9/64	0.5/1.8
IC_{50} hERG (µM)	>30	-	-

Supplementary Table 4 | Pharmacokinetics of compounds **20** and **21** after i.v. administration to CD-1 male mice.

Parameter	Compound **20**[a]	Compound **21**[a]
i.v. dose (mg/kg)	1.0	1.0
Matrix collected	Plasma	Blood
t1/2 (hr)	0.51	0.79
Cl (mL/min/kg)	174	27.5
AUCinf (hr*ng/mL)	95.9	606
Vd (L/kg)	7.2	1.5
MRT (hr)	0.69	0.89

[a] Mean of n =3

References

1. Kruse, T. *et al.* Mechanisms of site-specific dephosphorylation and kinase opposition imposed by PP2A regulatory subunits. *EMBO J* **39**, e103695 (2020).
2. Cundell, M. J. *et al.* A PP2A-B55 recognition signal controls substrate dephosphorylation kinetics during mitotic exit. *J Cell Biol* **214**, 539–54 (2016).
3. Hein, J. B. *et al.* Phosphatase specificity principles uncovered by MRBLE:Dephos and global substrate identification . *Mol Syst Biol* **19**, (2023).
