# Supplementary material for: The balance between B55α and Greatwall expression levels predicts sensitivity to Greatwall inhibition in cancer cells
Source: Nat Commun. 2025 Aug 27;16:8016. doi: 10.1038/s41467-025-62943-z (PMC12391391; doi:10.1038/s41467-025-62943-z)
Supplement: Supplementary file 2 — Reporting Summary [file 41467_2025_62943_MOESM2_ESM.pdf]

nature portfolio

Corresponding author(s): Róbert Zach
Helfrid Hochegger

Last updated by author(s): Jul 21, 2025

Reporting Summary

Nature Portfolio wishes to improve the reproducibility of the work that we publish. This form provides structure for consistency and transparency in reporting. For further information on Nature Portfolio policies, see our Editorial Policies and the Editorial Policy Checklist.

Statistics

For all statistical analyses, confirm that the following items are present in the figure legend, table legend, main text, or Methods section.

n/a	Confirmed	
☐	☒	The exact sample size (n) for each experimental group/condition, given as a discrete number and unit of measurement
☐	☒	A statement on whether measurements were taken from distinct samples or whether the same sample was measured repeatedly
☐	☒	The statistical test(s) used AND whether they are one- or two-sided<br>*Only common tests should be described solely by name; describe more complex techniques in the Methods section.*
☒	☐	A description of all covariates tested
☐	☒	A description of any assumptions or corrections, such as tests of normality and adjustment for multiple comparisons
☐	☒	A full description of the statistical parameters including central tendency (e.g. means) or other basic estimates (e.g. regression coefficient) AND variation (e.g. standard deviation) or associated estimates of uncertainty (e.g. confidence intervals)
☐	☒	For null hypothesis testing, the test statistic (e.g. F, t, r) with confidence intervals, effect sizes, degrees of freedom and P value noted<br>*Give P values as exact values whenever suitable.*
☒	☐	For Bayesian analysis, information on the choice of priors and Markov chain Monte Carlo settings
☒	☐	For hierarchical and complex designs, identification of the appropriate level for tests and full reporting of outcomes
☐	☒	Estimates of effect sizes (e.g. Cohen's d, Pearson's r), indicating how they were calculated

Our web collection on statistics for biologists contains articles on many of the points above.

Software and code

Policy information about availability of computer code

Data collection	Raw immunofluorescence imaging data were stored on the OMERO cloud repository. Cells and nuclei were segmented using Cellpose (version 2.0.5) and custom-built segmentation models as part of a custom-made automated image analysis pipeline (https://github.com/HocheggerLab/Omero_Screen.git). Phospho-peptides were identified using Mascot (v2.8.0.1) and quantified using Pescal.
Data analysis	Analysis of differentially phosphorylated peptides was performed in R (version 4.2.3) using custom scripts (https://github.com/R-Zach/Cellular-responses-to-Greatwall-inhibition.git). Downstream image analysis and quantifications were done using Python (version 3.9.13) or R (version 4.2.3) and custom-built scripts (https://github.com/R-Zach/Cellular-responses-to-Greatwall-inhibition.git). In mathematical modeling of C-604 sensitivity, linear regression and model fitting were performed with the fitdistrplus package (version 1.2-1) in R (version 4.4.0). In structural modeling of C-604 binding to the active site of GWL, molecular graphic figures were prepared using PyMOL (v3.10).

For manuscripts utilizing custom algorithms or software that are central to the research but not yet described in published literature, software must be made available to editors and reviewers. We strongly encourage code deposition in a community repository (e.g. GitHub). See the Nature Portfolio guidelines for submitting code & software for further information.

Data

Policy information about availability of data

All manuscripts must include a data availability statement. This statement should provide the following information, where applicable:

- Accession codes, unique identifiers, or web links for publicly available datasets
- A description of any restrictions on data availability
- For clinical datasets or third party data, please ensure that the statement adheres to our policy

The mass spectrometry proteomics data have been deposited to the ProteomeXchange Consortium via the PRIDE partner repository with the dataset identifier PXD059047 and 10.6019/PXD059047.

Additional data files can be obtained from the following repositories:
https://doi.org/10.6084/m9.figshare.28829927
https://doi.org/10.6084/m9.figshare.28829816
https://doi.org/10.6084/m9.figshare.28829915
https://doi.org/10.6084/m9.figshare.28829903
https://doi.org/10.6084/m9.figshare.28829306
https://doi.org/10.6084/m9.figshare.28848074

Research involving human participants, their data, or biological material

Policy information about studies with human participants or human data. See also policy information about sex, gender (identity/presentation), and sexual orientation and race, ethnicity and racism.

Reporting on sex and gender	*Use the terms sex (biological attribute) and gender (shaped by social and cultural circumstances) carefully in order to avoid confusing both terms. Indicate if findings apply to only one sex or gender; describe whether sex and gender were considered in study design; whether sex and/or gender was determined based on self-reporting or assigned and methods used.* *Provide in the source data disaggregated sex and gender data, where this information has been collected, and if consent has been obtained for sharing of individual-level data; provide overall numbers in this Reporting Summary. Please state if this information has not been collected.* *Report sex- and gender-based analyses where performed, justify reasons for lack of sex- and gender-based analysis.*
Reporting on race, ethnicity, or other socially relevant groupings	*Please specify the socially constructed or socially relevant categorization variable(s) used in your manuscript and explain why they were used. Please note that such variables should not be used as proxies for other socially constructed/relevant variables (for example, race or ethnicity should not be used as a proxy for socioeconomic status).* *Provide clear definitions of the relevant terms used, how they were provided (by the participants/respondents, the researchers, or third parties), and the method(s) used to classify people into the different categories (e.g. self-report, census or administrative data, social media data, etc.)* *Please provide details about how you controlled for confounding variables in your analyses.*
Population characteristics	*Describe the covariate-relevant population characteristics of the human research participants (e.g. age, genotypic information, past and current diagnosis and treatment categories). If you filled out the behavioural & social sciences study design questions and have nothing to add here, write "See above."*
Recruitment	*Describe how participants were recruited. Outline any potential self-selection bias or other biases that may be present and how these are likely to impact results.*
Ethics oversight	*Identify the organization(s) that approved the study protocol.*

Note that full information on the approval of the study protocol must also be provided in the manuscript.

Field-specific reporting

Please select the one below that is the best fit for your research. If you are not sure, read the appropriate sections before making your selection.

☒ Life sciences ☐ Behavioural & social sciences ☐ Ecological, evolutionary & environmental sciences

For a reference copy of the document with all sections, see nature.com/documents/nr-reporting-summary-flat.pdf

Life sciences study design

All studies must disclose on these points even when the disclosure is negative.

Sample size	In most experiments, the sample size was defined either by the number of experimental replicates ($n \geq 3$) or by the number of cells that were imaged and segmented using automated imaging and image processing. For live-cell imaging analysis, 10 distinct cell lineages were generated for each cell line and condition, as this number was sufficient to capture population-level characteristics. For UMAP analysis, 500 cells were randomly selected per experiment, cell line, and condition, as this sample size adequately represented the overall population characteristics.

Data exclusions	No data were excluded from the analyses.
Replication	All findings were successfully replicated.
Randomization	Not relevant; randomization was not used.
Blinding	Blinding was not applicable, as experiments were quantified using an automated analysis or conducted by a single person.

Reporting for specific materials, systems and methods

We require information from authors about some types of materials, experimental systems and methods used in many studies. Here, indicate whether each material, system or method listed is relevant to your study. If you are not sure if a list item applies to your research, read the appropriate section before selecting a response.

Materials & experimental systems

n/a	Involved in the study	
☐	☒	Antibodies
☐	☒	Eukaryotic cell lines
☒	☐	Palaeontology and archaeology
☒	☐	Animals and other organisms
☒	☐	Clinical data
☒	☐	Dual use research of concern
☒	☐	Plants

Methods

n/a	Involved in the study	
☒	☐	ChIP-seq
☒	☐	Flow cytometry
☒	☐	MRI-based neuroimaging

Antibodies

Antibodies used	anti-GWL (Sigma-Aldrich, HPA027175), anti-PPP2R2A (Santa Cruz, sc-81606), anti-ENSA/ARPP19 (Abcam, ab180513), anti-ENSA (pS67)/ARPP19(pS62) (CST, 5240), anti-PPP2C (CST, 2038), anti-β-actin (Abcam, ab3280), anti-GAPDH (Abcam, ab8245), anti-6His (Clontech, 631212), anti-phospho-CDK substrate motif [(K/H)pSP] (CST, 9477S), anti-mouse HRP-fused (Dako, P0447), anti-rabbit HRP-fused (Dako, P0448), anti-α-tubulin (CST, 3873), anti-p21 (CST, 2947), anti-mouse Alexa Fluor 488 conjugate (CST, 4408S), anti-rabbit Alexa Fluor 555 conjugate (CST, 4413S)
Validation	All antibodies were validated by the manufacturer. Anti-GWL (Sigma-Aldrich, HPA027175) and anti-PPP2R2A (Santa Cruz, sc-81606) antibodies were validated by western blot using protein extracts from cells transfected with siRNAs targeting GWL or PPP2R2A. Anti-ENSA(pS67)/ARPP19(pS62) (CST, 5240) antibody was validated by western blot using protein extracts from asynchronous and prometaphase-arrested cells. Anti-α-tubulin (CST, 3873) and anti-p21 (CST, 2947) antibodies were validated by immunofluorescence microscopy.

Eukaryotic cell lines

Policy information about cell lines and Sex and Gender in Research

Cell line source(s)	Cell lines were provided by the GDSC Research Tissue Bank.
Authentication	Cell lines were validated by ATCC STR.V service.
Mycoplasma contamination	All cell lines were negative for mycoplasma contamination. Mycoplasma testing was carried out using the Lonza MycoAlert® Detection Kit.
Commonly misidentified lines (See ICLAC register)	*Name any commonly misidentified cell lines used in the study and provide a rationale for their use.*

Plants

Seed stocks	*Report on the source of all seed stocks or other plant material used. If applicable, state the seed stock centre and catalogue number. If plant specimens were collected from the field, describe the collection location, date and sampling procedures.*
Novel plant genotypes	*Describe the methods by which all novel plant genotypes were produced. This includes those generated by transgenic approaches, gene editing, chemical/radiation-based mutagenesis and hybridization. For transgenic lines, describe the transformation method, the number of independent lines analyzed and the generation upon which experiments were performed. For gene-edited lines, describe the editor used, the endogenous sequence targeted for editing, the targeting guide RNA sequence (if applicable) and how the editor was applied.*
Authentication	*Describe any authentication procedures for each seed stock used or novel genotype generated. Describe any experiments used to assess the effect of a mutation and, where applicable, how potential secondary effects (e.g. second site T-DNA insertions, mosiacism, off-target gene editing) were examined.*
